# Biocide-Tolerant Listeria monocytogenes Isolates from German Food Production Plants Do Not Show Cross-Resistance to Clinically Relevant Antibiotics

**DOI:** 10.1128/AEM.01253-19

**Published:** 2019-10-01

**Authors:** A. Roedel, R. Dieckmann, H. Brendebach, J. A. Hammerl, S. Kleta, M. Noll, S. Al Dahouk, S. Vincze

**Affiliations:** a German Federal Institute for Risk Assessment, Berlin, Germany; b University of Applied Sciences and Arts, Institute for Bioanalysis, Coburg, Germany; Rutgers, The State University of New Jersey

**Keywords:** *Listeria monocytogenes*, antibiotic resistance, biocide susceptibility, virulence factors

## Abstract

Contamination of food during processing is recognized as a main transmission route of Listeria monocytogenes. To prevent microbial contamination, biocides are widely applied as disinfectants in food processing plants. However, there are concerns about the development of antimicrobial resistance in foodborne pathogens due to widespread biocide usage. In our study, 93 L. monocytogenes isolates from German food production facilities were (i) tested for biocide and antibiotic susceptibility using broth microdilution assays, (ii) analyzed for links between reduced biocide susceptibility and antibiotic resistance, and (iii) characterized by whole-genome sequencing, including the detection of genes coding for biocide tolerance, antibiotic resistance, and other virulence factors. Fifteen L. monocytogenes isolates were tolerant to benzalkonium chloride (BAC), and genes conferring BAC tolerance were found in 13 of them. Antibiotic resistance was not associated with biocide tolerance. BAC-tolerant isolates were assigned to 6 multilocus sequence type (MLST) clonal complexes, and most of them harbored internalin A pseudogenes with premature stop codons or deletions (*n* = 9). Our study demonstrated a high genetic diversity among the investigated isolates including genotypes that are frequently involved in human infections. Although *in vitro* adaptation studies to biocides have raised concerns about increasing cross-resistance to antibiotics, our results do not provide evidence for this phenomenon in field isolates.

**IMPORTANCE** Foodborne pathogens such as L. monocytogenes can persist in food production environments for a long time, causing perennial outbreaks. Hence, bacterial pathogens are able to survive cleaning and disinfection procedures. Accordingly, they may be repeatedly exposed to sublethal concentrations of disinfectants, which might result in bacterial adaptation to these biocides. Furthermore, antibiotic coresistance and cross-resistance are known to evolve under biocide selection pressure *in vitro*. Hence, antimicrobial tolerance seems to play a crucial role in the resilience and persistence of foodborne pathogens in the food chain and might reduce therapeutic options in infectious diseases.

## Abstract (precis)

Foodborne pathogens such as L. monocytogenes can persist in food production environments for a long time, causing perennial outbreaks. Hence, bacterial pathogens are able to survive cleaning and disinfection procedures. Accordingly, they may be repeatedly exposed to sublethal concentrations of disinfectants, which might result in bacterial adaptation to these biocides. Furthermore, antibiotic coresistance and cross-resistance are known to evolve under biocide selection pressure *in vitro*. Hence, antimicrobial tolerance seems to play a crucial role in the resilience and persistence of foodborne pathogens in the food chain and might reduce therapeutic options in infectious diseases.

## INTRODUCTION

Listeriosis is one of the most serious foodborne diseases. Despite the low incidence of listeriosis (0.47 cases per 100,000 population, 2016, European Union), the high hospitalization (98%) and case fatality rate (16.2%) compared to other zoonotic agents render it a serious public health concern (1). The causative agent, Listeria monocytogenes, is a Gram-positive, facultative intracellular opportunistic pathogen. Human infections with L. monocytogenes predominantly occur after the consumption of contaminated ready-to-eat food products (2). The ubiquitous microorganism may contaminate a wide range of foodstuffs during the various steps of food production and distribution (2). To fulfill hygiene requirements according to EC regulation no. 852/2004 on the hygiene of foodstuffs (3), biocides are widely applied as disinfectants to prevent bacterial contamination. In Germany, the Industrial Hygiene and Surface Protection Association (Industrieverband Hygiene & Oberflächenschutz [IHO]) maintains a list of disinfectants that have been tested according to German (DIN; German Institute for Standardization) and European (EN) standards for use in the health care sector, in animal husbandry, and in food production (https://www.iho.de/). They include quaternary ammonium compounds (QACs), aldehydes, alcohols, chlorine-releasing compounds, or peracids. The awareness of risks related to subinhibitory biocide concentrations triggering antimicrobial resistance in bacteria has substantially increased in the last years (4, 5). In *in vitro* experiments, links between reduced biocidal susceptibility and antibiotic resistance have been described for various substances and bacterial species (6–11), including L. monocytogenes (12, 13). Biocide tolerance may be based on similar resistance mechanisms toward different antimicrobial agents (cross-resistance). In the case of coresistance, the mechanisms conferring reduced susceptibility are unrelated but genetically linked, e.g., located on the same genetic element (14). However, the relevance of co- and cross-resistance has not yet been validated in the environment and therefore needs to be verified in field studies.

So far, standardized laboratory methods to investigate biocide susceptibility are not available, and harmonized breakpoints defining biocide tolerance are also lacking. Tolerance is defined as reduced susceptibility of bacteria toward a biocide characterized by a raised MIC (5). Determining epidemiological cutoffs (ECOFFs) for MICs and minimum bactericidal concentrations (MBCs) help interpret susceptibility profiles in a bacterial population. Currently, ECOFF data for biocides are limited to a few bacterial species (15, 16) but do not include L. monocytogenes. Epidemiological studies on biocide susceptibility mainly focused on the determination of MICs of QACs (17–19). MIC values provide only limited information on tolerance to in-use concentrations of disinfectants. Hence, MICs can only be interpreted as trend indicators for reduced biocide susceptibility. In addition, MBC values should be determined to evaluate lethality of the in-use concentration of a biocide (5).

Increased tolerance against antimicrobial stress triggered by the application of disinfectants may be an important factor for the persistence of L. monocytogenes in food production environments (20, 21). Particularly, members of the small multidrug resistance (SMR) protein family are associated with reduced susceptibility to quaternary ammonium compounds like benzalkonium chloride (BAC). The SMR transporter genes identified in L. monocytogenes are *qacH* (22), *emrE* (23), *emrC* (24), and the *bcrABC* cassette (25). The *bcrABC* cassette consists of a transcriptional regulator gene, *bcrA*, and two SMR genes (*bcrB* and *bcrC*). In addition, enhanced expression of efflux pump genes belonging to the major facilitator superfamily (MFS), such as *mdrL*, can contribute to BAC tolerance (26).

To the best of our knowledge, data on the biocide susceptibilities of L. monocytogenes isolates originating from Germany are not available, and a link between biocide tolerance and antibiotic resistance in L. monocytogenes has not yet been proven. We assume that the selection pressure in food processing plants is high because of the widespread use of disinfectants in hygiene processes. The aim of our study was to examine the biocide susceptibilities of L. monocytogenes isolates from food production plants in Germany and to look for potential relationships between biocide tolerance and antibiotic resistance. We therefore tested the susceptibilities to six antimicrobial biocides frequently used in the food industry and to antibiotics relevant for human listeriosis therapy. Further, we analyzed the genetic diversity of the L. monocytogenes strains under study and investigated the prevalence of putative biocide tolerance and antibiotic resistance genes as well as virulence genes.

## RESULTS

### Phenotypic analysis.

**FIG 1:**
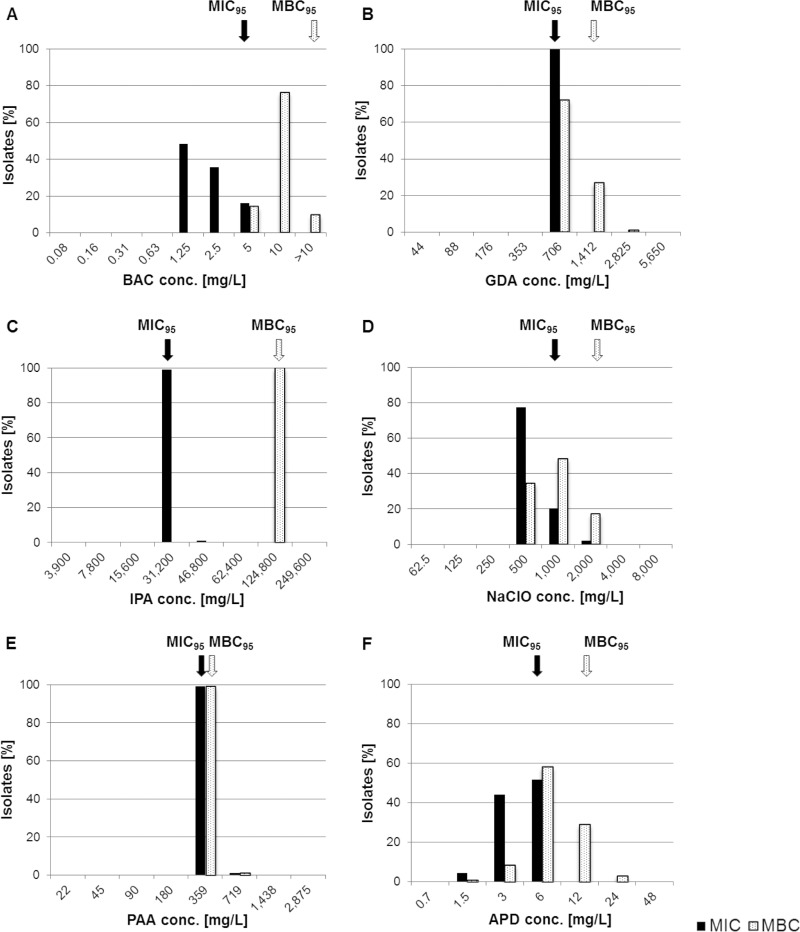
MIC (black bars) and MBC (white bars) distributions of 93 L. monocytogenes isolates. Arrows mark MIC_95_ (black) and MBC_95_ (white) values representing tentative ECOFFs. conc., concentration; ECOFF, epidemiological cutoff; BAC, benzalkonium chloride; GDA, glutaraldehyde; IPA, isopropanol; NaClO, sodium hypochlorite; PAA, peracetic acid; APD, biocidal product containing bis(3-aminopropyl)dodecylamine.

**(i) Susceptibility to biocides.** In pretests, the neutralizer used for MBC evaluation proved to be effective for all six biocides under investigation and revealed no toxicity (data not shown). An overview on the results of biocide susceptibility testing (MIC and MBC) is given in Fig. 1. MIC and MBC data were not normally distributed. Narrow unimodal MIC and MBC distributions ranging between one and three dilution steps were observed for all biocides. The only exception was bis(3-aminopropyl)dodecylamine (APD), showing a broader MBC distribution of five dilution steps.

Tentative ECOFFs were empirically set, and isolates with reduced susceptibility toward the tested biocides revealed MIC and/or MBC values above the ECOFFs. Elevated MICs were found for isopropanol (IPA; *n* = 1) and sodium hypochlorite (NaClO; *n* = 2) (Fig. 1). Increased MBCs were detected for glutaraldehyde (GDA; *n* = 1) and APD (*n* = 3). One isolate showed both MIC and MBC values above the ECOFFs for peroxyacetic acid (PAA).

Applying the predefined MIC breakpoint for BAC (≥4 mg/liter) published previously (26, 27), 16% of the isolates (*n* = 15) were classified as BAC tolerant.

**(ii) Antibiotic susceptibility testing.** All isolates were daptomycin (DPT) resistant but ampicillin (AMP), penicillin G (PEN), vancomycin (VAN), erythromycin (ERY), gentamicin (GEN), linezolid (LIZ), tetracycline (TET), and trimethoprim-sulfamethoxazole (T/S) sensitive. Variable susceptibility patterns were observed for tigecycline (TGC; resistance [R], 76%), meropenem (MER; R, 8%), ciprofloxacin (CIP; susceptible, increased exposure [I], 5%), and rifampin (RAM; I, 1%) (see Table S1 in the supplemental material).

Five isolates (5%) were resistant to three different classes of antibiotics and therefore defined as multidrug resistant. Antibiotic resistance profiles did not differ significantly between biocide-tolerant and -susceptible isolates (*P* > 0.05). Spearman correlation coefficients revealed no association between biocide tolerance and antibiotic resistance (data not shown).

### Genotypic characterization.

**FIG 2:**
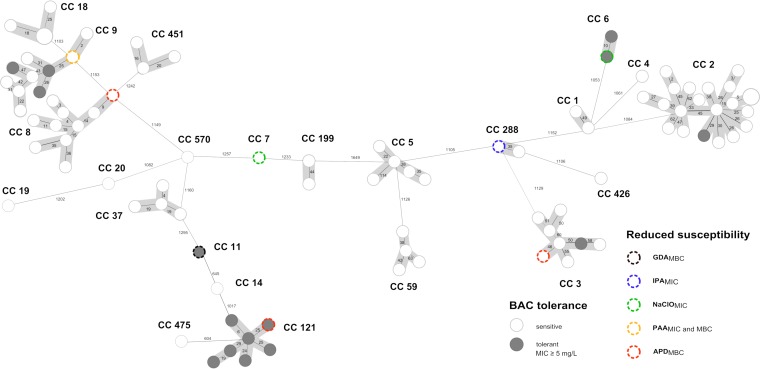
Minimum-spanning tree based on core genome MLST (cgMLST) allelic profiles of 93 L. monocytogenes isolates from food production environments in Germany. Each circle represents an allelic profile derived from sequence analysis of 1,701 cgMLST target genes. The size of each circle corresponds to the number of isolates. Numbers on the connecting lines illustrate numbers of target genes with differing alleles in a pairwise comparison. Isolates with reduced biocide susceptibility are color-coded as specified in the legend. Allelic patterns belonging to identical MLST clonal complexes (CC) are shaded in gray. BAC, benzalkonium chloride; GDA, glutaraldehyde; IPA, isopropanol; NaClO, sodium hypochlorite; PAA, peracetic acid; APD, biocidal product containing bis(3-aminopropyl)dodecylamine.

**(i) Genetic diversity of L. monocytogenes.** Core genome multilocus sequence typing (cgMLST) revealed a broad genetic diversity among L. monocytogenes isolates from food production environments (Fig. 2). According to classical multilocus sequence typing (MLST), 23 sequence types (STs) belonging to 23 distinct clonal complexes (CCs) were identified. CC2 (23% [*n* = 21]), CC8 (11% [*n* = 10]), CC9 (11% [*n* = 10]), CC3 (9% [*n* = 8]), and CC121 (8% [*n* = 7]) were the most prevalent clonal complexes within this study.

Phenotypically, all isolates belonging to CC121 (*n* = 7), CC6 (*n* = 2), and CC11 (*n* = 1) were BAC tolerant. CC2, CC3, and CC9 isolates were either BAC susceptible or tolerant. Three out of 15 BAC-tolerant isolates also showed reduced susceptibility to NaClO (CC6), GDA (CC11), or APD (CC121).

**FIG 3:**
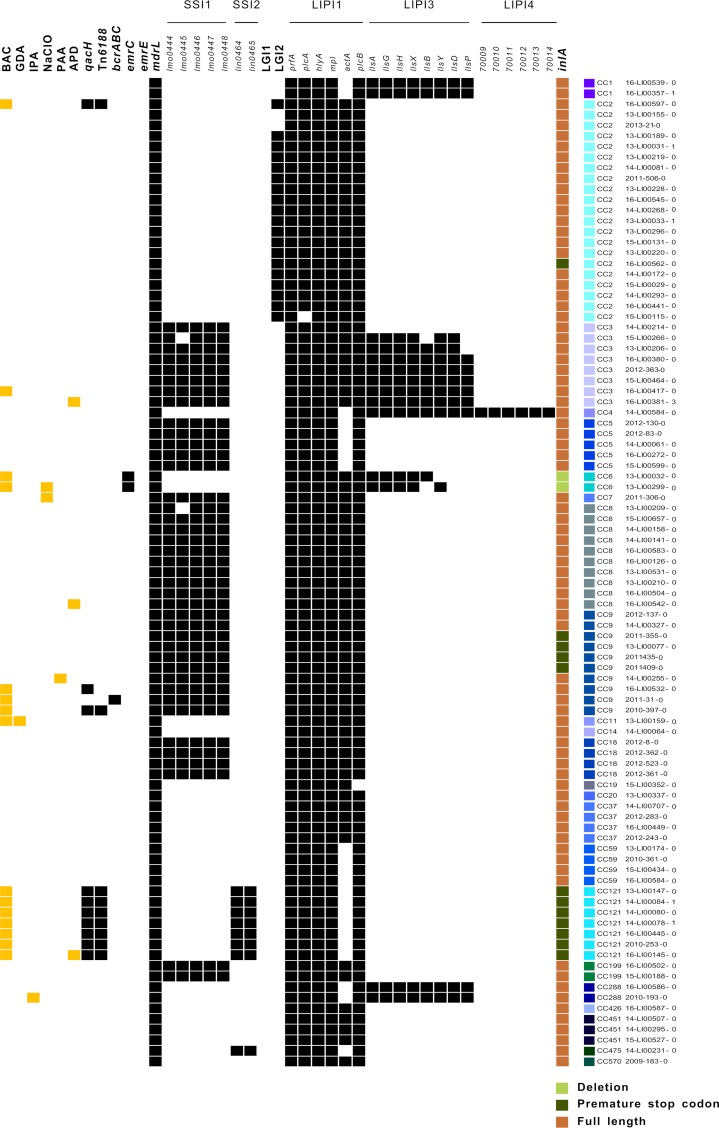
Distribution of biocide tolerance and virulence genes. Listeria monocytogenes isolates with reduced susceptibility to biocides are highlighted in yellow. The occurrence of genetic determinants is shown in black. Deletions and truncations of *inlA* are shaded green, whereas a full-length *inlA* is brown. BAC, benzalkonium chloride; GDA, glutaraldehyde; IPA, isopropanol; NaClO, sodium hypochlorite; PAA, peracetic acid; APD, biocidal product containing bis(3-aminopropyl)dodecylamine; SSI, stress survival islet; LIPI, *Listeria* pathogenicity island; LGI, *Listeria* genomic island; CC, clonal complex.

**(ii) Detection of genes conferring biocide tolerance.** In order to detect major determinants of reduced susceptibility or tolerance to biocides in the study population, we screened the translated amino acid sequences against the BacMet database. Five amino acid sequence motifs were exclusively present in BAC-tolerant isolates, whereas isolates tolerant to other biocides did not reveal unique determinants (Table 1). Four of the identified motifs belonged to SMR efflux transporters. Subsequently, the genomes of all isolates were screened for the presence of genes corresponding to the five amino acid sequence motifs, including *qacH*, *bcrABC*, and *emrC* (Fig. 3). *qacH* encoding an SMR efflux transporter was present in 10 out of 15 BAC-tolerant isolates. Single-nucleotide polymorphism (SNP) analysis revealed high similarities between nine of these genes and previously described *qacH* on the nucleotide level (GenBank accession no. HF565366.1) (22). In these cases, *qacH* was located on transposon Tn*6188* (Fig. 3). One isolate, however, carried a gene with 92% similarity to *qacH* but lacked the transposon. According to blastn analysis, the gene was identical to a sequence of an uncultured organism (GenBank accession no. KJ792090).

**TABLE 1:** Unique biocide tolerance genes of BAC-tolerant L. monocytogenes isolates

BacMet database protein^*a*^	Species	UniProt accession no.	blastn similarity to Listeria monocytogenes NCBI:txid1639				Sample ID by clonal complex^*b*^														
							CC2	CC3	CC6		CC9			CC11	CC121						
							16-LI00597-0	16-LI00417-0	13-LI00032-0	13-LI00299-0	16-LI00532-0	2010-397-0	2011-31-0	13-LI00159-0	13-LI00147-0	14-LI00078-1	14-LI00080-0	14-LI00084-1	16-LI00145-0	16-LI00445-0	2010-253-0
			Corresponding gene	GenBank accession no.	Query cover (%)	Identity (%)															
BcrA	L. monocytogenes	I7B1B9	*bcrA*	JX023284.1	16–100	80–100	x	-	-	-	x	x	x	-	x	x	x	x	x	x	x
BcrB	L. monocytogenes	I7A797	*bcrB*	JX023284.1	100	100	-	-	-	-	-	-	x	-	-	-	-	-	-	-	-
BcrC	L. monocytogenes	I6ZWM1	*bcrC*	JX023284.1	100	99	-	-	-	-	-	-	x	-	-	-	-	-	-	-	-
Multidrug resistance protein	Enterococcus faecalis	Q82YU7	*emrC*	LT732640.1	100	100	-	-	x	x	-	-	-	-	-	-	-	-	-	-	-
QAC resistance protein QacC	S. aureus	P14319	*qacH*	HG329628.1	100	92–100	x	-	-	-	x	x	-	-	x	x	x	x	x	x	x

a QAC, quaternary ammonium compound.

b ID, identification. x, present; -, absent.

Both BAC-tolerant CC6 isolates carried *emrC*, a gene encoding another SMR efflux transporter. The *bcrABC* cassette, encoding BcrA, BcrB, and BcrC, was detected in one BAC-tolerant isolate belonging to CC9. The BcrA regulator was found in 10 more isolates lacking BcrBC. The detection of the complete *bcrA* gene sequence was limited to the *bcrABC* cassette carrying a CC9 isolate. The regulator sequences of the remaining 10 isolates revealed only small segments (sequence query coverages, 16 to 33%) with high similarities (≥80%) compared to *bcrA*. The regulator was located upstream of *qacH* in all 10 genomes and showed 85 to 100% similarity to *tetR* family transcriptional regulator genes.

We also screened for other genes that are known to convey BAC tolerance. The *emrE* gene, coding for an SMR efflux pump, could not be detected in our study population. However, the nonspecific efflux pump gene *mdrL* was present in all isolates tested.

**(iii) Detection of antimicrobial resistance genes.** In whole-genome sequencing (WGS) data, only the fosfomycin resistance gene *fosX* was detected, which was present in all isolates. Analysis of genes that can confer resistance to carbapenems (genes coding for penicillin-binding proteins [PBPs]) or to TGC (*rpsJ*) due to point mutations did not reveal any alterations in the sequence structure that have been previously linked to resistance.

**(iv) Detection of virulence genes.** We looked for various Listeria-specific virulence factors in the tested study population (Fig. 3). The stress survival islets 1 (SSI-1) and SSI-2 were identified in 43% (*n* = 40) and 9% (*n* = 8) of the isolates, respectively. SSI-2 was significantly more often identified in BAC-tolerant isolates than in susceptible isolates (*P* < 0.001). None of the L. monocytogenes isolates harbored the *Listeria* genomic island 1 (LG1). In contrast, LG2 was identified in 19 isolates of CC2.

Genes coding for *Listeria* pathogenicity island 1 (LIPI-1) were highly conserved in the study population. While none of the isolates harbored LIPI-2, which is a species-specific pathogenicity island of Listeria ivanovii, LIPI-3 was detected in 15% (*n* = 14) of the isolates belonging to CC1, CC3, CC4, CC6, and CC288. LIPI-4 was found in one isolate of CC4.

A full-length internalin A (*inlA*) gene was detected in 85% of the isolates. Most of the BAC-tolerant isolates (*n* = 9) harbored *inlA* genes with premature stop codons or deletions. While all isolates of CC121, four CC9 isolates, and one CC2 isolate harbored *inlA* genes with premature stop codons, both CC6 BAC-tolerant isolates showed a 9-bp deletion.

## DISCUSSION

The consumption of contaminated food is the primary source of human listeriosis. Listeria monocytogenes can survive harsh conditions in food production facilities, such as low temperature, acidic environments, and disinfection procedures. Thus, contamination of food in the production process is recognized as a major transmission pathway (2). To obtain deeper insight into the properties of L. monocytogenes from German food production facilities, we investigated (i) biocide susceptibilities for frequently used substances in food processing plants, (ii) putative associations between reduced susceptibility to biocides and antibiotic resistance, and (iii) the genetic diversity, with a special focus on virulence factors, antibiotic resistance, and biocide tolerance.

### Biocide susceptibility.

Increased tolerance of L. monocytogenes to biocides used in disinfection measures appears to contribute to pathogen persistence, as previously shown for BAC (17, 21). Beside BAC, we examined five additional substances relevant for food hygiene with the aim to broaden the knowledge on the biocide susceptibility of L. monocytogenes. Although some isolates showed slightly increased MIC and/or MBC values to several biocides (Fig. 1), the resistance of these isolates under in-use concentrations is unlikely, because the MBC values measured were below the concentrations applied during disinfection (https://www.iho.de/). We determined tentative ECOFFs for all investigated substances to distinguish between susceptible isolates and isolates with reduced susceptibility. In our study, MIC and MBC values were not normally distributed, indicating the need for an increased number and diversity of isolates to be investigated in order to define more reliable ECOFFs. The tentative ECOFFs we defined reveal various limitations because of the lack of standardized biocide susceptibility testing methods, the small sample size investigated, and the fact that ECOFFs typically refer to normally distributed populations (28).

Previous studies applied a MIC breakpoint of ≥4 mg/liter to differentiate BAC-susceptible from -tolerant L. monocytogenes (26, 27). According to this definition, a high percentage of the isolates under study (16%) would be considered tolerant. Epidemiological studies from Switzerland and Norway reported similar prevalences of BAC-tolerant L. monocytogenes (29, 30). Higher rates ranging from 46% to 79% were observed in Turkey and Spain (31, 32). These data highlight the need for regular surveillance of biocide susceptibility, especially in the case of disinfectants widely used in food production facilities. Since November 2016, BAC has been listed as an unapproved disinfectant and preservative in the European Union (implementation decision 2016/1950). In the future, the reduced BAC application may lead to a decrease in the prevalence of BAC-tolerant L. monocytogenes isolates.

It proves difficult to compare epidemiological studies because of the variety of breakpoints defined for BAC tolerance (≥4 mg/liter up to 20 mg/liter) (17, 18, 21). In our study, we were able to show that 13 out of 15 (87%) L. monocytogenes isolates with MIC values of ≥5 mg/liter harbor genes which are known to contribute to BAC tolerance, such as *qacH*, *emrC*, and *bcrABC*.

The majority of BAC-tolerant isolates harbored the *qacH* gene located on the transposon Tn*6188*. In previous studies, *qacH* on Tn*6188* was predominantly found in isolates belonging to CC121 and CC9 (18, 21, 32) but was also reported in CC2 (21), which is in line with our results. Interestingly, one CC9 isolate carried a *qacH* gene that was not located on Tn*6188*. Alignment of the sequences revealed 92% similarity to Tn*6188*-carried *qacH* genes. This study reports L. monocytogenes harboring *qacH* in the absence of Tn*6188*.

The efflux transporter gene *emrC* was just recently identified in L. monocytogenes isolates belonging to CC6 (24). Kremer and colleagues further proved an association between reduced BAC susceptibility due to *emrC* and increased MICs for amoxicillin and gentamicin. In our study, *emrC* was detected in two CC6 isolates, but reduced antibiotic susceptibility was not observed, suggesting that the presence of *emrC* is not necessarily associated with antibiotic resistance. One BAC-tolerant CC9 isolate carried the *bcrABC* cassette (Fig. 2), which has been described before (21). In the two isolates which did not carry unique biocide tolerance genes, reduced susceptibility might have been induced by the overexpression of endogenous efflux pump genes, like *mdrL*, belonging to the MFS family (26).

Besides the known BAC tolerance genes, mechanisms have been described that might contribute to reduced susceptibility to NaClO, QACs, and PAA (27, 33, 34). In this context, biofilm formation or modifications of cell surface properties by alteration of membrane fatty acids and phospholipids that inhibit biocides to enter the cell have been discussed (27). Further, the glutamate decarboxylase system is well known as an acid tolerance system in L. monocytogenes (33). To what extent these mechanisms contribute to reduced susceptibility to NaClO, PAA, and other substances tested in our study needs to be elucidated.

### Antibiotic susceptibility and cross-resistance.

The fact that biocides like disinfectants can be a driver for antibiotic resistance becomes more and more a concern in the scientific community (5). *In vitro* studies demonstrated an association between biocide tolerance and reduced susceptibility to antibiotics in L. monocytogenes (12, 13). In our study, biocide tolerance and antibiotic resistance did not correlate, indicating that the mechanisms responsible for the determined BAC tolerance do not necessarily lead to cross-resistance to the tested antibiotics. Overall, antibiotic susceptibility profiles revealed a low level of resistance in L. monocytogenes isolated from food production environments in Germany. However, it is alarming that 8% of the isolates in our study were resistant to meropenem because this carbapenem may be used as alternative therapy for bacterial meningitis (35). In an epidemiological study from Poland, the prevalence of meropenem resistance (40%) in L. monocytogenes isolates from fish processing plants was even higher (36). In contrast, other studies did not detect meropenem resistance at all among isolates from meat processing plants or human patients (37, 38). In Gram-positive bacteria, carbapenem resistance can be associated with mutation-derived changes in their PBPs (39) which we could not detect in the meropenem-resistant isolates of our study.

All isolates tested were resistant to daptomycin, which is in line with the results from a previous study in our National Reference Laboratory focusing on food isolates (40). However, there are reports that described a lower prevalence of daptomycin resistance in L. monocytogenes (41, 42). Nevertheless, daptomycin cannot be recommended for the treatment of human listeriosis because of the reduced susceptibility of L. monocytogenes (42).

So far, daptomycin resistance mechanisms of *Listeria* spp. are not fully understood. Other Gram-positive bacteria like Staphylococcus aureus, Enterococcus spp., and Streptococcus spp., developed various strategies to counteract daptomycin, which mainly involve adaptive changes in the cell wall and cell membrane homeostasis (reviewed by Tran et al. [43]).

Resistance to tigecycline was very common in our study population, which was associated neither with the presence of *tetL* and *tetM* genes nor with mutations in *rpsJ*, resistance determinants that have been described for other Gram-positive bacteria (44, 45). The overexpression of unspecific efflux pumps can also be responsible for tigecycline resistance (46). In previous studies, tigecycline-resistant L. monocytogenes isolates were found in lower numbers (40, 41).

### Genotypic diversity.

Molecular typing of L. monocytogenes is essential in order to detect disease clusters and to identify food-related sources of infection as early as possible. Pulsed-field gel electrophoresis, the former gold standard for isolate differentiation in outbreak investigations, is increasingly replaced by WGS-based typing methods (47). In this way, the spatial and temporal distribution of L. monocytogenes genotypes can be compared. Our data revealed a broad heterogeneity of L. monocytogenes MLST clonal complexes in the food production environment, with CC2, CC8, and CC9 as predominant genotypes. In Germany, CC8 and CC2 isolates are frequently reported as causative agents of human listeriosis (48, 49). However, we also identified many isolates that belonged to genotypes of minor clinical importance in Germany, e.g., CC9 and CC121, which were defined as food-associated genotypes (50, 51). Due to limited sample access, our strain collection does not provide comprehensive information on the nationwide prevalence of L. monocytogenes genotypes in German food production facilities.

### Identification of virulence and stress response genes.

Listeria monocytogenes is a heterogeneous species displaying various degrees of virulence (51). The ability of L. monocytogenes to survive harsh environmental conditions is enhanced in isolates carrying SSIs (52, 53). SSI-1 supports survival under acidic conditions and high salt concentrations (53) and can be found equally in isolates from humans, food, and food processing environments (52). Accordingly, we identified isolates of various clonal complexes that carried SSI-1. SSI-2 contributes to the survival of L. monocytogenes under alkaline and oxidative stress (52) and is predominantly found in ST121 isolates (belonging to CC121) (52, 54), which is in line with our results. Even though SSI-2 was significantly more frequently identified in BAC-tolerant isolates, Harter and colleagues were able to show that this gene cluster does not mediate tolerance to QACs (52).

LGIs have been associated with increased virulence, heavy metal resistance, and BAC tolerance (23, 55, 56). In our study, isolates only carried LGI2. LGI2 codes for genes involved in pathogenicity and arsenic resistance and seems to be widely present in clinical isolates belonging to CC1, CC2, and CC4 (56). We consistently detected LGI2 in most CC2 isolates (90%).

Carriage of LIPIs promotes virulence. LIPI-1, a pathogenicity island modulating host cell functions, is highly conserved in L. monocytogenes (57), and parts of this gene cluster were omnipresent in our isolate collection. LIPI-3 codes for a hemolytic and cytotoxic factor that impacts virulence and is associated with several clonal complexes, including CC1, CC4, and CC6 (58–60). We detected LIPI-3 in all isolates belonging to CC1 and in the single CC4 isolate. In addition, this pathogenicity island was present in all CC3, CC6, and CC288 isolates. We found LIPI-4 only in the single CC4 isolate of our study. LIPI-4 was recently identified in clinical L. monocytogenes isolates of CC4 and is linked to hypervirulence (51).

The *inlA* gene codes for a protein that is involved in the invasion of human intestinal epithelial cells and is considered an important virulence factor of L. monocytogenes. Premature stop codons resulting in the truncation of *inlA* are associated with attenuated virulence. They are predominantly detected in nonhuman isolates (61). Consistent with previous findings (51), all CC121 and several CC9 isolates (40%) from our study harbored truncated *inlA* genes. Franciosa et al. showed that isolates with a truncated *inlA* gene displayed increased capacity for biofilm formation (62), which may be associated with biocide tolerance and persistence properties.

Listeria monocytogenes from German food production facilities obviously carried virulence factors contributing to human infection. While some of the genes known to be involved in virulence were present in all or most of the isolates under study, others only occurred in specific clonal complexes.

### Conclusion.

Our study revealed a high genetic diversity among L. monocytogenes isolates from technical equipment and surfaces of German food production facilities. The detection of genotypes that are frequently involved in human listeriosis highlights the importance of contaminated food production environments as transmission routes for virulent L. monocytogenes. Phenotypic tolerance to BAC was observed in 15 isolates (16%), and efflux pump genes conferring BAC tolerance were identified in 13 of them. Exposure to low concentrations of quaternary ammonium compounds can occur as a result of improper disinfection practices and may enhance the ability of selected isolates to persist in niches within food production environments. However, given the low overall prevalence of biocide-tolerant isolates, it is likely that additional factors contribute to the persistence of L. monocytogenes, including the ability to form biofilms.

BAC tolerance and the presence of BAC tolerance genes were not associated with antibiotic resistance, indicating that the mechanisms responsible for reduced BAC susceptibility in the investigated isolates do not confer antibiotic resistance. Moreover, most of the BAC-tolerant isolates harbored internalin A pseudogenes which are known to occur in isolates that exhibit reduced virulence and enhanced biofilm-forming ability. Altogether, our study does not support significant associations between biocidal selective pressure in food production and antimicrobial tolerance of L. monocytogenes. However, from *in vitro* studies, we know that links between biocide tolerance and antibiotic resistance do exist in bacteria. The widespread use of disinfectants might therefore lead to a selection of antibiotic-resistant isolates and needs regular monitoring. Last but not least, a better understanding of the phenotypic traits that contribute to the survival and persistence of L. monocytogenes in food processing plants and their underlying genetic determinants is required and a prerequisite for infection control of listeriosis.

## MATERIALS AND METHODS

### Listeria monocytogenes isolates.

Ninety-three L. monocytogenes isolates, collected by official food control authorities from 2008 through 2016 in German food production plants and archived at the National Reference Laboratory for L. monocytogenes (Germany), were characterized (Table S1). The isolates originated from various surfaces of food processing facilities and equipment, such as slicers, cutting boards, handles, sinks, grinders, cutting tables, derinders, gutters, tubes, and floor drains. Species identification was carried out by biochemical and molecular typing, as previously described (40). Isolates were stored at −80°C until use. Isolates were chosen under consideration of the source and year of isolation, with the main aim of including a highly diverse study population.

### Biocides.

Susceptibility of the L. monocytogenes isolates was tested to six biocides commonly used to sanitize food contact surfaces, namely, the quaternary ammonium compound BAC (≥95%; Sigma-Aldrich, Steinheim, Germany), GDA (50%; Carl Roth, Karlsruhe, Germany), IPA (≥99.9%; Carl Roth), the chlorine-releasing compound NaClO (12% Cl, techn.; Carl Roth), the oxidizing agent PAA (36 to 40% [wt/vol]; Sigma-Aldrich), and a biocidal product (Budenat Intense D443; Buzil-Werk Wagner, Memmingen, Germany) containing APD (7.5% [wt/wt]) as an active ingredient. The biocides were serially diluted in 2-fold steps just before the experiment using standardized hard water as defined in EN 1276, as follows: 10 to 0.08 mg/liter BAC, 5,650 to 44 mg/liter GDA, 249,600 to 3,900 mg/liter IPA, 8,000 to 62.5 mg/liter free chlorine (NaClO), 2,875 to 22 mg/liter PAA, and 48 to 0.7 mg/liter APD in Budenat.

### Biocide susceptibility testing.

**(i) MICs.** The MICs of the biocides under study were determined by broth microdilution assays. An overnight culture of each isolate grown on tryptic soy agar (TSA; Merck, Darmstadt, Germany) was adjusted to about 10^6^ CFU/ml 2-fold concentrated tryptic soy broth (TSB; Merck). In a 96-well microtiter plate (Greiner Bio-One, Frickenhausen, Germany), 50 μl of the bacterial solution was added to 50 μl of the double-concentrated biocide. The plate was incubated at 37°C for 20 ± 2 h. Optical density at 595 nm (OD_595_) was measured after 5 s of shaking using the Mithras^2^ multimode reader (Berthold Technologies, Bad Wildbad, Germany; Software MikroWin 2010 v5.18, German UI). Bacterial growth was compared to a negative control (microtiter well containing biocide solution and TSB), and a ΔOD_595_ of 0.1 was considered the cutoff value. The MIC was defined as the lowest concentration of a biocide at which no growth was observed. Biological replicates derived from two independent experiments were conducted on different dates. A MIC variation of one dilution step between the two experiments was accepted. The lower value was defined as the MIC. In case of higher variation, the test was repeated once more, and the median was considered the final MIC.

### (ii) Minimum bactericidal concentration.

The MBC of each strain and biocide was determined by broth microdilution according to Knapp et al., with minor modifications (63). Dey-Engley neutralizing broth (Sigma-Aldrich) was used to quench biocidal effects for MBC testing. The neutralizer efficacy and toxicity were tested before according to Knapp et al. (64). The MBC was defined as the lowest concentration of the biocide which revealed no visible colonies on TSA.

### Determination of tentative ECOFFs.

According to EUCAST guidelines (28) tentative ECOFFs can be defined to distinguish between susceptible isolates and isolates with reduced antibiotic susceptibility. Following this approach, tentative ECOFFs of unimodal MIC or MBC distributions were defined for tested biocides at concentrations representing 95% of the bacterial population (MIC_95_ and MBC_95_, respectively).

### Antibiotic susceptibility testing.

Antibiotic susceptibilities (S) to AMP (S, ≤2 mg/liter), CIP (S, ≤1 mg/liter; R, ≥4 mg/liter), DPT (S, ≤1 mg/liter), ERY (S, ≤0.5 mg/liter; R, ≥8 mg/liter), GEN (S, ≤4 mg/liter; R, ≥16 mg/liter), LIZ (S, ≤4 mg/liter; R, ≥8 mg/liter), MER (S, ≤0.25 mg/liter), PEN (S, ≤2 mg/liter), RAM (S, ≤1 mg/liter; R, ≥4 mg/liter), TET (S, ≤4 mg/liter; R, ≥16 mg/liter), TGC (S, ≤0.5 mg/liter; R, >0.5 mg/liter), T/S (S_T/S_, ≤0.05/9.5 mg/liter), and VAN (S, ≤2 mg/liter; R, ≥16 mg/liter) were determined using the commercial test system Micronaut S *Listeria* MHK-2 (Merlin Gesellschaft für Mikrobiologische Diagnostika mbH, Bornheim, Germany), as previously described (40). Resistance was assessed using clinical breakpoint guidelines of the Clinical and Laboratory Standards Institute (CLSI) (65, 66). If no breakpoints for L. monocytogenes were available, those recommended for Staphylococcus spp. were applied. Since CLSI breakpoints for tigecycline were missing, cutoffs defined by the European Committee on Antimicrobial Susceptibility Testing (EUCAST) were used (67).

### Statistical analysis.

Spearman rank coefficients (Rho) were calculated to investigate the correlation of MICs or MBCs between tested biocides and antibiotics using SPSS (IBM SPSS Statistics, v21; IBM Corp., Armonk, NY, USA). Data were tested for normal distribution by the Kolmogorov-Smirnov test. For comparative analysis between two groups of isolates (biocide sensitive versus biocide tolerant), the Mann-Whitney test was applied. *P* values of <0.05 were considered to be significant.

### Next-generation sequencing.

Listeria monocytogenes isolates were cultivated on sheep blood agar (SBA). A single colony was transferred into brain heart infusion (BHI) bouillon and incubated at 37°C for 18 to 20 h while shaking (150 rpm). DNA was extracted from bacterial cells using the PureLink genomic DNA minikit (Invitrogen, Carlsbad, CA, USA). WGS libraries were prepared with the Nextera XT DNA sample preparation kit (Illumina, San Diego, CA, USA), according to the manufacturer’s protocol. Paired-end sequencing (2 × 301 cycles) was performed with the MiSeq reagent v3 600-cycle kit (Illumina) on an Illumina MiSeq benchtop sequencer.

First, sequence read quality was analyzed with FastQC v0.11.5 (Babraham Bioinformatics, Cambridge, United Kingdom). Second, sequence reads were assembled using SPAdes v3.10.0 with the options BayesHammer read error correction, postprocessing mismatch corrector with BWA, and an automatic coverage filter (68). Third, assembly quality was analyzed using Quast v4.5 by comparison to the L. monocytogenes type strain EGD-e (NCBI:txid169963, NCBI RefSeq accession no. NC_003210.1).

### Classical MLST and cgMLST.

For phylogenetic comparison of the L. monocytogenes isolates, classical MLST and cgMLST were performed on the basis of WGS data. Classical MLST and corresponding clonal complexes were determined according to the scheme of the Institut Pasteur (https://bigsdb.pasteur.fr/listeria/). cgMLST analysis was carried out using the Ridom SeqSphere+ software (v4.0.1; Ridom GmbH, Münster, Germany), according to Ruppitsch et al. (69). The cgMLST scheme relies on a set of 1,701 target genes that are present in >99% of the known genomes of the species. The combination of all alleles in a strain forms a profile that can be used to characterize the phylogenetic relationships among isolates.

### *In silico* screening for biocide resistance determinants on protein level.

WGS data of the L. monocytogenes isolates under study were screened for the presence of experimentally confirmed resistance proteins recorded in the BacMet database (70) (Antibacterial Biocide and Metal Resistance Genes database, http://bacmet.biomedicine.gu.se/, BacMet v2, last updated 9 December 2017).

The rapid prokaryotic genome annotation software Prokka v1.12 (71) was used to delimit open reading frames (ORFs) in the draft genomes and to annotate protein-coding genes by hierarchical feature prediction at the amino acid sequence level with BLAST+ v2.6.0 and HMMER v3.1b2.

The BacMet database of “experimentally confirmed resistance genes” included 753 amino acid sequences which were uploaded into Prokka as a user-provided set of annotated proteins for the initial round of feature prediction. The annotation of the most significant match (E value, >31) within the BacMet database was transferred to an ORF. BacMet-flagged *Listeria* ORFs were counted and summarized in a genome/feature table for subsequent correlation with phenotypic data (Table S1).

### Analysis of biocide tolerance determinants and virulence factors on nucleotide level.

Comparative analyses of genes conferring biocide tolerance were carried out using the BioNumerics software v7.6.2 (Applied Maths, Sint-Martens-Latem, Belgium).

We analyzed genes coding for SMR efflux transporters, i.e., *qacH* on the transposon Tn*6188* (GenBank accession no. HF565366), *emrC* (GenBank accession no. LT732640.1), *emrE* (GenBank accession no. CP001602), *bcrABC* (GenBank accession no. JX023284.1), and *mdrL* (GenBank accession no. AJ012115.1) coding for an efflux pump belonging to the MFS.

Furthermore, we looked for the following virulence factors: SSI-1 (GenBank accession no. NC_003210) and SSI-2 (NC_003212.1), LGI1 (CP001602) and LGI2 (CM001159.1), and the *Listeria* pathogenicity islands (LIPI-1, AL591974.1; LIPI-2, AJ004808.1; LIPI-3, AE017262.2; and LIPI-4, CYWW02000024.1). Additionally, the coding sequence for *inlA* (NC_003210) was investigated to determine whether isolates possess a full-length gene, deletions, or truncated sequences indicated by a premature stop codon. A minimum % sequence identity (%ID) threshold of 80% and a minimum length of 80% of the target gene were used for sequence identification.

### Investigation of antibiotic resistance genes.

Acquired antibiotic resistance determinants were identified by ResFinder 3.0 (Center for Genomic Epidemiology; http://www.genomicepidemiology.org/) (72). Listeria monocytogenes penicillin binding protein genes *lmo1892*, *lmo2039*, *lmo1438*, *lmo2229*, *lmo0441*, *lmo2754*, *lmo0540*, *lmo1916*, *lmo1855*, and *lmo2812* (NCBI RefSeq accession no. NC_003210) were analyzed for single-nucleotide polymorphisms which might contribute to meropenem resistance (73, 74). Furthermore, the *rpsJ* gene (NCBI RefSeq accession no. NC_003210) was analyzed for point mutations, which have been previously associated with TGC resistance in Enterococcus faecium (44).

### Data availability.

The sequences of three representative *qacH* genes of 16-LI00597-0, 13-LI00147-0, and 16-LI00532-0 were deposited in the National Center for Biotechnology Information database (https://www.ncbi.nlm.nih.gov/) under accession numbers MK944275 to MK944277, respectively.

## Supplementary Material

Supplemental file 1
